# Influence of Vertebrobasilar Stenotic Lesion Rigidity on the Outcome of Angioplasty and Stenting

**DOI:** 10.1038/s41598-020-60906-6

**Published:** 2020-03-03

**Authors:** Feng-Chi Chang, Chao-Bao Luo, Chih-Ping Chung, Kuei-Hong Kuo, Ting-Yi Chen, Han-Jui Lee, Chung-Jung Lin, Jiing-Feng Lirng, Wan-Yuo Guo

**Affiliations:** 1 Department of Radiology, Taipei Veterans General Hospital, Taipei, Taiwan; 2 National Yang Ming University, School of Medicine, Taipei, Taiwan; 3 Department of Neurology, Section of Cerebrovascular Disease, Neurological Institute, Taipei Veterans General Hospital, Taipei, Taiwan; 4 Division of Medical Image, Far Eastern Memorial Hospital, New Taipei City, Taiwan

**Keywords:** Interventional cardiology, Stroke

## Abstract

Stenotic lesion rigidity (SLR) has an unclear influence on the outcome of percutaneous transluminal angioplasty and stenting (PTAS) for intracranial arterial stenosis. This study evaluated the outcome of PTAS and the relationship of vertebrobasilar SLR to features on vessel wall MRI (VW-MRI) for identifying pathologies of vertebrobasilar stenosis (VBS) and evaluating PTAS outcome. We retrospectively evaluated the results of PTAS in 31 patients with severe VBS. Stenotic lesions were classified as soft (based on predilatation pressure [PP] ≦ 4 atm) in 15 patients or hard (PP >4 atm) in 16 patients. We examined the relationship of SLR to clinical and MR findings. Patients with hard vs soft lesions had atherosclerosis (8/16 [50.0%] vs 2/15 [13.3%]), dissection (0/16 [0.0%] vs 12/15 [80.0%]), and dissection in atherosclerosis (8/16 [50.0%] vs 1/15 [6.7%], P < 0.0001); high intensity signal on the T1WI of VW-MRI (5/16 [31.3%] vs 14/15 [93.3%]) and iso- to low intensity signal (11/16 [68.7%] vs 1/15 [6.7], P = 0.001), and significant in-stent restenosis (>50%) in 5/15 (33.3%) vs 0/15 (0.0%) (P = 0.0421) in the 30 patients who successfully completed PTAS. Vertebrobasilar SLR correlated well with lesion etiology, findings on VW-MRI, and PTAS outcome. Patients with hard stenotic lesions need close follow-up after PTAS.

## Introduction

Vessel wall-MR imaging (VW-MRI) has recently been used to diagnose the etiology of intracranial arterial stenosis by direct visualization of the vessel wall^1,2^. Although the application of VW-MRI enhances the accuracy of determining the etiology of vertebrobasilar stenosis (VBS), some challenges remain. First, various pathologies of VBS share similar morphological and signal characteristics on VW-MRI. Second, stenotic vascular lesions can have mixed pathologic features, making definitive diagnosis difficult to determine^3^. Medical treatment outcomes are worse if vertebrobasilar dissection is associated with atherosclerotic intracranial arteries^4^. However, diagnosis is unclear when there is a mixed pathology, i.e., when a dissecting lesion occurs in an arterial segment with atherosclerotic changes. There is also no report describing the relationship of stenotic lesion rigidity (SLR) to the outcome of endovascular management of VBS caused by vessel wall dissection in an atherosclerotic artery.

In the SAMMPRIS trial, the incidence of procedure-related vessel wall rupture was 1.8% in the stenting group^5,6^. Using a high balloon pressure to dilate hard or vulnerable stenotic lesions may account for this devastating complication. However, insufficient predilatation of severely stenotic lesions can result in significant residual stenosis and increase difficulty of stent deployment or the risk of delayed in-stent restenosis (ISR)^7^. This suggests that the vertebrobasilar SLR is an important factor in the selection of patients for percutaneous transluminal angioplasty and stenting (PTAS). This retrospective study was designed to examine the relationship of vertebrobasilar SLR to MR findings that provide diagnostic clues to pathologies of VBS and can be used to evaluate the outcome of PTAS.

## Materials and Methods

This retrospective study was approved by the Institutional Review Board of Taipei Veterans General Hospital. All procedures performed in the studies involving human participants were in accordance with the ethical standards of the institutional and/or national research committee and with the 1964 Helsinki Declaration and relevant HIPAA guidelines. To perform the MR exam and endovascular procedure, pre-procedural informed consents were obtained from patients or, when the patients were under age 18 years, from their parents and/or legal guardians.

### Patients

**Figure 1:**
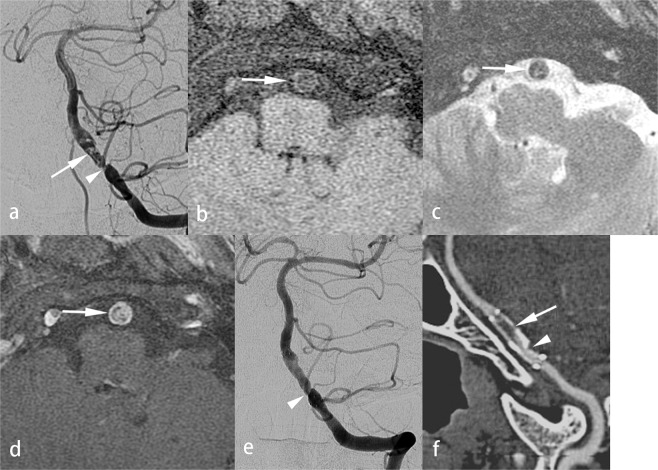
Hard lesion in a patient with dissection in atherosclerosis (DA). A 54-year-old man was a heavy smoker and had a history of neck massage. He suffered from stroke with right hemiparesis in the past 1 year and had recurrent acute diplopia and dysarthria 1 month ago. Left vertebral angiogram showed a 90% stenotic lesion of the left distal vertebral artery (**a**, arrowhead) and disrupted intimal flaps (**a**, arrow). VW-MRI showed low signal intensity on fat-suppressed T1WI (**b**, arrow) and T2WI (**c**, arrow) and strong enhancement on contrast-enhanced fat-suppressed T1WI (**d**, arrow). PTAS was done and revealed a predilatation pressure (PP) of 6.5 atm. A 4 × 20 mm Wingspan stent was deployed in the left distal vertebral artery to basilar artery (**e**). About 30% residual stenosis was noted on the control angiogram (**e**, arrow). A 2-year follow-up CTA, curved multi-planar reconstruction, revealed 90% in-stent restenosis (**f**, arrow) and 30% residual stenosis of the proximal stent (**f**, arrowhead).

Between 2011 to 2018, thirty-one patients (28 males [90.3%], 3 females [9.7%]; mean age 62.2 ± 13.9 [16–85] years old) with severe symptomatic VBS, who had PTAS, were enrolled in this study (Table 1, supplementary information). All patients received medical treatment on presentation of symptoms including impaired consciousness due to cerebral ischemia in the vertebrobasilar territory. Clinical and MR findings^1^ were used to identify the etiology of the vascular stenosis as either atherosclerosis, dissection, or dissection in atherosclerosis (DA)^8^. Dissection was diagnosed by clinical history of traumatic events (such as massage) or headache and MR findings of intramural hematoma. DA was diagnosed by the presence of dissection in an atherosclerotic segment of an artery (Fig. 1). Besides having clinical findings of atherosclerosis and dissection, patients with DA can present with intimal dissection on angiography, or calcified plaques on CT or MRI, or both. Atherosclerosis-related risk factors were also recorded. The indications for PTAS in our cases of VBS were: 1) more than 70% arterial stenosis on imaging study; 2) medically refractory neurological symptoms, including rapidly deteriorated consciousness level. PTAS was performed on an urgent basis when neurological symptoms were recurrent and rapidly becoming more severe.

**Table 1:** Demographic features and clinical findings of the 31 patients with severe, symptomatic vertebrobasilar stenosis who underwent angioplasty and stenting.

Variable	
Age, years	62.2 ± 13.9 (16–85)
Sex: male/female	28 (90.3%)/3 (9.7%)
Risk factors	
Hypertension	19 (61.3%)
Diabetes	10 (32.3%)
Hyperlipidemia	9 (29.0%)
Smoking	16 (51.6%)
Diagnosis	
Atherosclerosis	10 (32.3%)
Dissection	12 (38.7%)
Dissection in atherosclerosis	9 (29.0%)
Indication for PTAS	
TIA	6 (19.4%)
Stroke	25 (80.6%)
Time interval between symptoms and MR exam (mon)	4.48 ± 7.24 (0.03–30)
MR findings	
Brain infarcts on MRI	
Nil	5 (16.1%)
Brainstem	19 (61.3%)
Cerebellum	17 (54.8%)
Thalamus/occipital region	17 (54.8%)
Associated other intracranial arterial stenosis	
Nil	16 (51.6%)
ICA	13 (41.9%)
MCA	7 (22.6%)
ACA	1 (3.2%)
Location of stenotic lesions	
Basilar artery	15 (48.4%)
Vertebral artery	4 (12.9%)
Vertebral to basilar artery	12 (38.7%)
Severity of stenotic lesions (%)	84.0 ± 10.2 (60–99)
Signals of the most stenotic lesions on HR-VWI	
T2WI: high/iso-to low	7 (22.6%)/24 (77.4%)
T1WI: high/iso-to low	19 (61.3%)/12 (38.7%)
T1WI + C: strong/faint or no	10 (32.3%)/21 (67.7%)
Restricted diffusion of the stenotic lesions:yes/no	7 (22.6%)/24 (77.4%)
Angioplasty and stenting	
Predilatation pressure (atm)	4.7 ± 1.8 (2–7)
Stents (self-expandable/balloon-expandable, n = 30)	20 (66.7%)/10 (33.3%)
Technical success	30 (96.8%)
Complications	11 (35.4%)
Outcomes (n = 30)	
Follow-up period (mon)	22.3 ± 17.4 (1–60)
Severe restenosis (≥50%)	5 (16.7%)
Recurrent stroke/symptoms	3 (10.0%)
Mortality	3 (10.0%)

T1WI + C = contrast-enhanced T1WI.

### MR examination

The MR machines were 1.5 T systems (Signa 1.5 T HDxt CVi & Discovery 1.5 T MR450, GEM, Waukesha, Wisconsin, USA). All patients underwent an MR examination within 3 days before PTAS. The MR examination included axial FLAIR and DWI of the brain, TOF MRA of the brain, contrast-enhanced MRA of the neck, and VW-MRI of the vertebrobasilar territory. The stenotic lesions of the vertebrobasilar territory visualized on TOF MRA were then investigated by VW-MRI. We used a 2-dimensional technique and took multi-planar images perpendicular to the long axis of the whole stenotic segment. The sequences included T1WI, T2WI, and contrast-enhanced T1WI. The protocol of VW-MRI was: slice thickness = 3 mm, NEX = 6 for T2WI and NEX = 4 for T1WI, FOV = 13 × 13 cm. Fat suppression and blood flow suppression techniques were applied^1^.

On VW-MRI, the relative overall plaque signal intensity of the vascular wall of the stenotic segment was compared with the signal intensity of the nearby medial pterygoid muscles^9^. High signal intensity was defined as more than 150% of the signal intensity of the medial pterygoid muscles. The enhancement of T1WI after injection of contrast media was either strong (signal intensity similar to that of the venous structures) or faint or absent. DWI (b = 1000 s/mm^2^) and ADC maps of brain were also used to evaluate the stenotic lesion.

### Percutaneous transluminal angioplasty and stenting

All patients received dual antiplatelet premedication. The procedures were done under either local or general anesthesia depending on the clinical status of the patients. After an intravenous bolus of 3000 to 5000 IU of heparin, a 5 or 6 Fr. guiding catheter was placed in the distal V2 segment of the dominant side of the vertebral artery. Pre-dilatation of the stenotic lesions was done with a non-compliant coronary balloon (Emerge®, Boston Sci. Co, MA, USA). The diameters of the target vertebrobasilar arteries were quantitatively measured on angiograms. The diameter of the balloon was chosen to be about 80 to 90% of the diameter of the adjacent normal segment. The selected balloon was slowly inflated in about 1 to 3 minutes to achieve adequate predilatation for stent placement. During the predilatation, the waist of the balloon appeared at the stenotic lesion site in the angiographic roadmap image and disappeared when the stenotic lesion was fully dilated by the balloon’s inflation pressure. The predilatation pressure (PP) was defined as the pressure at which this waist disappears in the angiographic roadmap image. The maximal pressure did not exceed 7 atm, even if the stenotic lesion was not fully dilated, and was used to classify SLR severity^5^. Stenotic lesions were characterized as hard (PP more than 4 atm) or soft (PP less than 4 atm). In contrast to maximal balloon pressure for predilatation of 6 atm used in the SAMMPRIS trial, it was 7 atm in our study because the plaques associated with vertebrobasilar stenosis were usually more densely calcified than the plaques in M1 of the MCA. Because 3 to 4 atm was the middle balloon pressure for predilatation in both the SAMMPRIS trial and the present study, we used 4 atm as the cut-off point between hard and soft lesions. After predilatation, the vessels were stented with either balloon-expandable stents (Apex®, Boston Sci Co, MA, USA) or self-expandable stents (Wingspan®, Boston Sci Co, Fremont, CA, USA). Balloon-expandable stents were not used in all cases because these self-expandable stents were not available at our institute before the end of 2012. Self-expandable stents are not covered by insurance in our country, making economic considerations a factor in the patient’s choice of balloon-expandable stents for their treatment. A control angiogram was performed to evaluate the residual stenosis. Any technical complications, including temporary symptoms of vertebrobasilar insufficiency, were recorded. All patients were followed up with clinical examination and CTA/MRA within the first post-procedural month and every 3–6 months after the treatment.

### Analysis

The clinical and MR findings and PTAS outcomes were compared between the soft and hard stenotic lesion groups (Table 2) and the clinical and MR characteristics and PTAS outcomes were compared between the dissection and DA groups (Table 3). The statistical analysis was performed using SPSS for Windows (version 18). For univariate analysis, differences in categorical variables were assessed using nonparametric methods, including the Fisher exact test, Wilcoxon two-sample test, and χ^2^ test. A *P*-value of 0.05 was considered a statistically significant difference.

**Table 2:** Comparison of hard and soft stenotic lesions in 31 patients with severe symptomatic vertebrobasilar stenosis who underwent angioplasty and stenting.

Variable	Hard lesions (n = 16)	Soft lesions (n = 15)	*P*	OR *(95% CI)*
Age, years	64.2 ± 8.9 (51–78)	60.1 ± 17.9 (16–85)	0.4172	
Sex (M/F)	13(81.3%)/3 (19.7%)	15 (100.0%)/0 (0.0%)	0.2258	
Risk factors				
Hypertension	13 (81.3%)	9 (60.0%)	0.2524	2.89 (0.57–14.68)
Diabetes	4 (25.0%)	6 (40.0%)	0.3719	0.50 (0.11–2.31)
Hyperlipidemia	8 (50.0%)	1 (6.7%)	0.0155	4.0 (1.47–133.23)
Smoking	8 (50.0%)	8 (53.3%)	0.8528	0.88 (0.21–3.58)
Diagnosis			<0.0001	
Atherosclerosis	8 (50.0%)	2 (13.3%)		1
Dissection	0 (0.0%)	12 (80.0%)		∞
Dissection in atherosclerosis	8 (50.0%)	1 (6.7%)		0.5 (0.04–6.68
Interval between symptoms and MR (mon)	5.30 ± 6.97 (0.1–24)	3.58 ± 8.00 (0.03–30)	0.0840	
MR findings				
Brain infarcts				
Brainstem	9 (56.3%)	10 (66.7%)	0.5518	0.64 (0.15–2.76)
Cerebellum	6 (37.5%)	11 (73.3%)	0.0732	0.22 (0.05–1.01)
Thalamus/occipital region	9 (56.3%)	8 (53.3%)	0.8700	1.13 (0.27–4.63)
Location of the stenotic lesions			0.8319	
Basilar artery	7 (43.8%)	8 (53.3%)		1
Vertebral artery	2 (12.5%)	2 (13.3%)		1.14 (0.13–10.39)
Vertebral to basilar artery	7 (43.8%)	5 (33.3%)		1.61 (0.35–7.40)
Severity of the stenosis (%)	83.6 ± 10.3 (70–99)	84.5 ± 10.4 (60–99)	0.6737	
Signals on HR-MRI				
T2WI: high/iso- to low	1 (6.3%)/15 (93.7%)	6 (40.0%)/9 (60.0%)	0.0373	0.1 (0.01–0.97)
T1WI: high/iso- to low	5 (31.3%)/11(68.7%)	14 (93.3%)/1 (6.7%)	0.0004	0.033 (0.003–0.32)
T1WI/C: strong/faint or no	4 (25.0%)/12(75.0%)	6 (40.0%)/9 (60.0%)	0.4578	0.50 (0.11–2.31)
Restricted diffusion of the stenotic lesions yes/no	1 (6.3%) /15 (93.7%)	6 (40.0%)/9 (60.0%)	0.0373	(0.01–0.97)
Stenosis of ICA & its branches	10 (62.5%)	5 (33.3%)	0.1044	(0.76–14.58)
Angioplasty and stenting				
Technical success	15 (93.7%)	15 (100.0%)	1.0000	−>0
Complications	6 (37.5%)	5 (33.3%)	0.8085	(0.27–5.25)
Stent type (self- /balloon- EX), n = 30	12 (80%)/3 (20%)	8 (53.3%)/7 (46.7%)	0.1213	(0.69–17.71)
Residual stenosis (%), n = 30	19.7 ± 23.2 (0.0–40.0)	11.3 ± 23.2 (0.0–30.0)	0.0070	—
Outcomes (n = 30)				
Follow-up period (mon)	18.9 ± 15.5 (1–55)	25.9 ± 19.1 (0.2–60)	0.2767	
Severe restenosis (≥50%)	5 (33.3%)	0 (0.0%)	0.0421	
Recurrent stroke/symptoms	3 (20.0%)	0 (0.0%)	0.2241	
Mortality	2 (13.3%)	1 (6.7%)	1.0000	

T1WI/C: contrast-enhanced T1WI; EX: expandable.

**Table 3:** Comparison of the stenotic lesions of vessels with dissection and dissection in atherosclerosis in 21 patients with severe symptomatic vertebrobasilar stenosis who underwent angioplasty and stenting.

Variable	Atherosclerosis (n = 10)	Dissection (n = 12)	Dissection in atherosclerosis (n = 9)	*P*	P*
Age, years	67.6 ± 20.1(62–80)	58.5 ± 19.2 (16–85)	61.2 ± 11.0 (50–78)	0.2197	0.8869
Sex (M/F)	8 (80%)/2 (20%)	12(100.0%)/0 (0.0%)	8 (88.9%)/1 (11.1%)	0.2828	0.4286
Risk factors					
Hypertension	10 (100%)	6 (50.0%)	6 (66.7%)	0.0345	0.6605
Diabetes	3 (30%)	4 (33.3%)	3 (33.3%)	0.9829	1.0
Hyperlipidemia	5 (50%)	1 (8.3%)	3 (33.3%)	0.0949	0.2722
Smoking	5 (50%)	5 (41.7%)	6 (66.7%)	0.5214	0.3870
Hardness of stenotic lesions				<0.0001	<0.0001
Hard	8 (80%)	0 (0.0%)	8 (88.9%)		
Soft	2 (20%)	12 (100.0%)	1 (11.1%)		
Interval between symptoms and MR (mon)	4.2 ± 8.3 (0.1–12)	4.2 ± 8.9 (0.03–30)	5.2 ± 8.0 (0.1–24)	0.2309	0.1628
MR findings					
Brain infarcts					
Brainstem	5 (50%)	8 (66.7%)	6 (66.7%)	0.6726	1.0
Cerebellum	5 (50%)	8 (66.7%)	4 (44.4%)	0.5585	0.3964
Thalamus/occipital region	6 (60%)	6 (50.0%)	5 (55.6%)	0.8945	0.8700
Location of the stenotic lesions				0.9704	0.8133
Basilar artery	5 (50%)	5 (41.7%)	5 (55.6%)		
Vertebral artery	1 (10%)	2 (16.6%)	1 (11.1%)		
Vertebral to basilar artery	4 (40%)	5 (41.7%)	3 (33.3%)		
Severity of the stenotic lesions (%)	84.4 ± 14.7(70–99)	85.3 ± 11.5 (60–99)	83.1 ± 11.1 (70–99)	0.7106	0.5648
Signals of the most stenotic lesions on HR-VWI					
T2WI: high/iso- to low	1 (10%)/9 (90%)	5 (41.7%)/7 (58.3%)	1 (11.1%)/8 (88.9%)	0.1298	0.1778
T1WI: high/iso- to low	3 (30%)/7 (70%)	11 (91.7%)/1(8.3%)	5 (55.6%)/4 (44.4%)	0.0116	0.1194
T1WI/C: strong/faint or no	0 (0%)/10 (100%)	6 (50.0%)/6(50.0%)	4 (44.4%)/5 (55.6%)	0.0287	1.0
Restricted diffusion of the stenotic lesions: yes/no	0 (0%)/10 (100%)	5 (41.7%) /7 (58.3%)	2 (22.2%)/7 (77.8%)	0.0666	0.6424
Stenosis of ICA & its branches	9(90%)	2 (16.7%)	4 (44.4%)	0.0027	0.3310
Angioplasty and stenting					
Predilatation pressure (atm)	5.9 ± 1.72 (2–7)	3.1 ± 0.5 (2.0–4.0)	5.7 ± 1.1 (4.0–7.0)	<0.0001	<0.0001
Technical success	9 (90%)	12 (100.0%)	9 (100.0%)	0.3379	1.0000
Complications	4 (40%)	4 (33.3%)	3 (33.3%)	0.9364	1.0
Stent type (self- /balloon- expandable)	8 (88.9%)/1 (11.1%), n = 9	5 (41.7%)/7 (58.3%)	7 (77.8%)/2 (22.2%)	0.0530	0.1842
Residual stenosis (%)	32.2 ± 8.3 (20–40), n = 9	13.3 ± 15.0 (0.0–30.0)	26.7 ± 14.1 (0.0–40.0)	0.0156	0.0618
Outcomes					
Follow-up period (mon)	19.2 ± 15 (3–48), n = 9	27.4 ± 18.6 (0.5–60)	19.0 ± 18.7 (1.0–55)	0.1108	0.1653
Severe restenosis (≥50%)	2 (22.2%), n = 9	0 (0.0%)	3 (33.3%)	0.2418	0.0632
Recurrent stroke/symptoms	1 (1.11%), n = 9	0 (0.0%)	2 (22.2%)	0.3094	0.1714
Mortality	1 (11.1%), n = 9	1 (8.3%)	1 (11.1%)	0.9696	1.000

*P*: comparison of atherosclerosis, dissection in atherosclerosis and dissection; P*: comparison of dissection in atherosclerosis and dissection; T1WI/C: contrast-enhanced T1WI.

### Informed consent

Informed consent was obtained from all participants included in the study.

## Results

All the results are shown in Tables 1–3.

### Patients

The demographic features, clinical and MR findings of the 31 patients with severely stenotic VBS lesions who received angioplasty and stenting are shown in Table 1. Atherosclerosis occurred in 10 cases (32.3%), dissection in 12 cases (38.7%), and DA in 9 cases (29.0%).

### MRI and VW-MRI

Restricted diffusion in the vertebrobasilar territory of the stenotic segment was noted on axial DWI of the brain in 7 patients (22.6%)^10^.

### Percutaneous transluminal angioplasty and stenting

**Figure 2:**
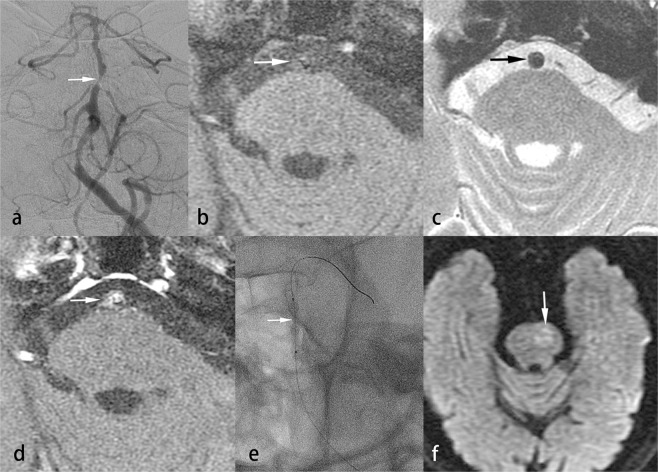
Hard lesion in a patient with atherosclerosis. A 73-year-old man suffered from recurrent stroke in the bilateral occipital regions. Left vertebral angiogram showed atherosclerosis with 90% stenosis of the mid-basilar artery (**a**, arrow). VW-MRI of the stenotic lesion of the mid-basilar artery revealed low signal on fat-suppressed T1WI (**b**, arrow) and T2WI (**c**, arrow) and strong enhancement on contrast-enhanced fat-suppressed T1WI (**d**, arrow). The PTAS failed because the stenotic lesion could not be fully dilated with the PP up to 7 atm (**e**, arrow). The Wingspan stent system could not pass through the stenotic lesion. His case was also complicated by a perforator infarct in the left pons (**f**, arrow).

PTAS was performed successfully in 30 of 31 patients (96.8%), under local anesthesia in 20 patients, and under general anesthesia in 11 patients. One patient failed stenting because the patient’s hard stenotic lesion could not be adequately dilated by an inflation pressure of 7 atm (Fig. 2). The Wingspan stent system failed to pass through the target lesion, leaving significant residual stenosis. The patient’s condition was complicated by slight dissection of the basilar artery and a perforator stroke due to pontine infarction. For all patients, the mean PP was 4.7 ± 1.8 (2–7) atm. Technical complications were noted in 11 patients (35.4%). They included perforator stroke in the brainstem of 7 (22.5%) patients and transient ischemic attack in 4 (12.9%) patients. In the 30 patients successfully treated with PTAS, recurrent neurological ischemia was noted in 3 of the 5 patients with severe ISR: 2 of the 3 patients died of a major stroke and one experienced a TIA.

### Statistical analysis of the hard and soft stenotic lesions

**Figure 3:**
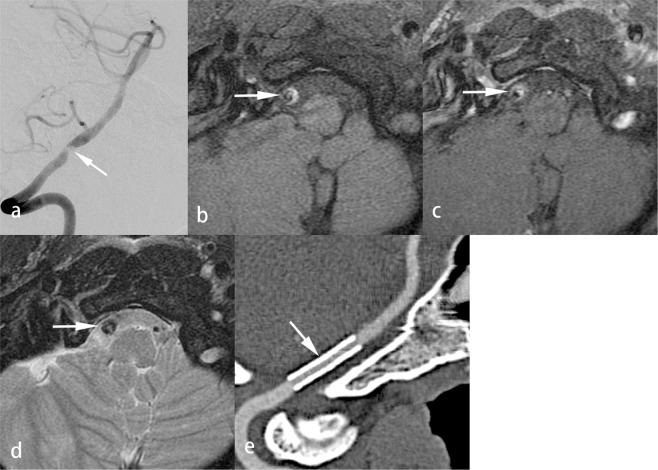
Soft lesion in a patient with dissection. A 44-year-old man suffered from recurrent headache and dysarthria for 1 week. Brain MRI showed multiple recent infarcts in the bilateral cerebellar hemispheres on DWI. Right vertebral angiogram showed acute dissection with 85% stenosis of the right distal vertebral artery (**a**). VW-MRI of the right distal vertebral artery showed a high-signal intramural hematoma on fat-suppressed T1WI (**b**, arrow) and T2WI (**c**, arrow) and focal strong enhancement of the intramural hematoma on contrast-enhanced fat-suppressed T1WI (**d**, arrow). During the PTAS, the PP was 3 atm. A 3.5 × 20 mm balloon-expandable stent was placed in the right distal vertebral artery. In a 4-year follow-up, CTA with curved multi-planar reconstruction showed good patency of the stent (**e**, arrow).

The clinical and MR results of the hard and soft lesion groups are shown in the Table 2. Patients in the hard lesion group more frequently had hyperlipidemia (8/16 [50%] vs 1/15 [6.7%], *P* = 0.0155) and a significantly higher PP (atm) (6.3 ± 0.9 [5.0–7.0] vs 3.1 ± 0.6 [2.0–4.0], *P* < 0.0001). The diagnosis of VBS was also significantly related to the hardness of the lesions (*P* < 0.0001). Hard lesions were noted in those with atherosclerosis and DA but not in those with dissection (Figs. 1, 2). For the soft lesion group, dissection was the most common diagnosis (12/15, 80%) (Fig. 3). On T1WI of VW-MRI, iso- to low intensity signals were significantly more frequent than high intensity signals in the hard lesion group (11/16 [68.7%] vs 5/16 [31.3%]) and high intensity signals were significantly more common than iso- to low signals in the soft lesion group (14/15 [93.3%] vs 1/15 [6.7%], *P* = 0.0004). On axial DWI of the brain, diffusion through the stenotic segment of the vertebrobasilar artery was significantly more restricted in the soft lesion group (5/15 [40.0%] vs 1/16 [6.3%], *P* = 0.0373).

Of the 30 patients successfully treated with PTAS, patients with hard lesions had significantly more residual stenosis on the control angiogram (19.7 ± 23.2 [0.0–40.0]% vs 11.3 ± 23.2 [0.0–30.0]%, *P* = 0.0070) and significantly more severe ISR (≥50%) (5/15[33.3%] vs 0/0[0.0%], *P* = 0.0421) during follow-up.

Table 3 lists the clinical and MR characteristics of the vertebrobasilar artery dissections in patients without atherosclerosis (the dissection group) and in patients with atherosclerosis (the DA group). The PP in the dissection and DA groups was 3.1 ± 0.5 (2.0–4.0) and 5.7 ± 1.1 (4.0–7.0), respectively (*P* < 0.0001). Although not statistically significant, there was a trend toward lower rate of residual stenosis (13.3 ± 15.0 [0.0–30.0%] vs 26.7 ± 14.1 [0.0–40.0%], *P* = 0.0618) and lower rate of stroke recurrence (0 [0.0%] vs 3 [33.3%], *P* = 0.0632) in the dissection group than in the DA group.

## Discussion

VW-MRI was originally developed to visualize the vessel wall directly, and it plays an important role in differential diagnosis, etiological diagnosis, and management planning^11^. However, VW-MRI has not been used to evaluate SLR in patients with intracranial arterial stenosis or to guide lesion management. The present study demonstrates the correlation between vertebrobasilar SLR and characteristics identified on MRI and VW-MRI. The soft vertebrobasilar lesions were mainly caused by dissection and had relatively high signal on T1WI with variable mural enhancement on VW-MRI. DWI of the brain also detected significantly more cases of restricted diffusion in lesions of the soft lesion group^10^. These results highlight the role of MRI in the pretreatment selection of patients with VBS. Using an endovascular procedure to evaluate SLR and to correlate SLR with clinical and MR findings provides an effective way to identify the etiology of vertebrobasilar stenosis.

Atherosclerotic lesion hardness has been reported to influence the outcome of endovascular management of peripheral arterial disease^12,13^. The hardness of plaques associated with carotid stenosis can also be accurately determined using a 1.5-T MR unit equipped with a neurovascular coil^14^. Our patients with soft lesions treated with PTAS had better clinical and imaging outcomes than those with hard lesions (Table 2). In the present study, soft stenotic lesions showed relatively high-intensity signals on fat-suppressed T1WI, which may suggest the presence of a hematoma in the arterial wall^15^. Other than intramural hematoma, soft plaques of carotid stenosis also reportedly show higher signal intensity on T1WI than non-soft plaques^16^. We suggest that PTAS, when technically safe to perform, is a good management option in “VBS with dissection” cases that show high signals on the T1WI of VW-MRI.

In the 183-patient stenting group of the SAMMPRIS trial, a 3-year ISR rate of 14.0% was noted^7^. During the 22.3 ± 17.4 months of follow-up in our study, severe ISR occurred in 5 patients, all of whom were in the hard lesion group (16.7%). All the hard stenotic lesions that we saw were in patients with atherosclerosis or DA. They commonly presented with iso- to low signals on both T1WI and T2WI of VW-MRI. These iso- to low signals on fat-suppressed T1WI and T2WI may suggest a lipid core or collagen or calcification^17^. Another possible explanation for the greater ISR severity in the hard lesion group is that the higher PP used in hard-lesion angioplasty causes greater intimal injury and tissue reaction. This suggests that patients with hard stenotic lesions should receive close post-procedural follow-up and more aggressive medical treatment. We also suggest the application of drug-eluting balloon angioplasty before stent placement in hard, stenotic, vertebrobasilar territory lesions to improve long-term outcome^18–20^.

Atherosclerotic disease is the most common cause of intracranial arterial stenosis. The clinical significance of VW-MRI findings in atherosclerosis has been described^1,21,22^. Dissection is a common etiology of VBS^23,24^ and an etiology of stroke, where it is more frequent in the posterior circulation territory than in the anterior circulation territory^25^. We correlated SLR with clinical and MR findings and proposed DA as a new diagnostic category (Fig. 1, Table 3). This new diagnosis applies to patients with stenotic lesions of mixed pathology in which the dissection occurs in a segment of stenosis caused by atherosclerosis. For DA, atherosclerotic plaques and an intramural hematoma in association with intimal surface injury are evident on VW-MRI or angiogram (Fig. 1). Intramural hematoma can be differentiated from intra-plaque hemorrhage by being localized within a plaque and not by being associated with intimal surface injury. Clinical history and symptoms of dissection, such as previous trauma, headache and neck pain, were found in our patients with DA. In Table 3, the clinical and MR findings were not significantly different between the DA group and dissection group. However, hard lesions were significantly more frequent in the DA group than the dissection group. This suggests that the signals of intramural hematomas in the DA and dissection groups were very similar on VW-MRI. Without a comprehensive neurological and neuroradiological evaluation, DA may be diagnosed as dissection, possibly delaying the process of stroke prevention.

The other clinical justifications for DA as a new disease entity include the following: (1) The complex pathology of DA may influence the temporal signal change of intramural hematomas at the dissection level on VW-MRI. The pathological status of plaques can change the process of regression and resorption of the adjacent intramural hematoma. Thus, the temporal change in signal intensity of the stenotic lesion in DA is not that of the stenotic lesion in dissection or atherosclerosis. For example, a chronic, unhealed dissection occurring in an inflammatory plaque may show low intensity signals on T1WI and enhancement after contrast injection on VW-MRI (Fig. 1). (2) Patients with DA have different outcomes from patients with dissection after endovascular management. Though not statistically significant in the present study, residual stenosis and ISR trended higher in the DA group than in the dissection group. Differentiating DA from dissection may help interventionists evaluate outcome. (3) Medical treatment outcomes differ between patients with DA and patients with dissection. The outcomes of medical treatment for dissection were worse in patients with neurovascular atherosclerosis than in patients without atherosclerosis^4^.

Endovascular management of VBS is associated with high risk of perforator stroke, which was noted in 7 (22.5%) of our cases. The higher incidence of neurological complications in our study than reported in the literature may be explained by our earlier treatment of symptoms within 7 days in some urgent cases, the technical difficulty of treating long irregular dissecting lesions, and the use of a rigorous protocol for evaluating events (ex. Some of our patients accepted PTAS under local anesthesia that enhanced detection of transient ischemic attack). The maximal PP of 7 atm used to dilate hard calcified plaques (higher than that used in the SAMMPRIS trial) may also increase the risk of vascular injury (Figs. 1 and 2). The above results suggest the importance of strictly following the indications or guidelines for endovascular management of intracranial arterial stenosis.

A limitation of this study is that multi-planar 2D acquisitions can result in partial averaging effects in curved arteries and less effective blood flow suppression than the 3D acquisition technique^1^. The very limited number of cases and the application of different stent types also made the analysis more difficult. Self-expandable stents are associated with higher risk of in-stent restenosis but also with lower technical risk and technical difficulty than the balloon-expandable stents. Although there was no statistical difference in the use of self-expandable and balloon-expandable stents between the 2 groups of this study, the lack of uniformity in stent types was still a limitation of this retrospective study. To elucidate the complex pathology and character of the arterial wall of intracranial arterial stenosis, we suggest adding high resolution imaging techniques, such as 3-dimensional VW-MRI performed with a high field MR scanner, vascular elastography, susceptibility weighted imaging, or MR-PET.

## Conclusions

Hard vertebrobasilar system lesions had significantly higher rate of residual stenosis and higher rate of ISR than soft lesions after PTAS. Patients with hard lesions, such as in cases of atherosclerosis and DA, as diagnosed by clinical and MR findings, need close follow-up after PTAS. The SLR of VBS correlated well with stenotic lesion etiologies and findings on VW-MRI and brain DWI-MRI. We used SLR as the basis for proposing DA as a new disease entity, which when compared with dissection, tended to be associated with higher rate of ISR. We suggest the use of VW-MRI of vertebrobasilar stenotic lesions for pre-procedural diagnosis and PTAS outcome evaluation. We also suggest adding advanced imaging techniques to elucidate the complex pathology of VBS.

## Supplementary information

Supplementary Information
