# Supplementary material for: Influence of Vertebrobasilar Stenotic Lesion Rigidity on the Outcome of Angioplasty and Stenting
Source: Sci Rep. 2020 Mar 3;10:3923. doi: 10.1038/s41598-020-60906-6 (PMC7054424; doi:10.1038/s41598-020-60906-6)
Supplement: Supplementary file 1 — Supplementary Information [file 41598_2020_60906_MOESM1_ESM.docx]

Supplementary Information

**Influence of Vertebrobasilar Stenotic Lesion Rigidity on the Outcome of Angioplasty and Stenting**

Feng-Chi Chang, MD^1,2^, Chao-Bao Luo, MD^1,2^, Chih-Ping Chung, MD, PhD^2,3^, Kuei-Hong Kuo, MD^2,4^, Ting-Yi Chen, BS^1,2^, Han-Jui Lee, MD^1,2^, Chung-Jung Lin, MD^1,2^, Jiing-Feng Lirng, MD^1,2^, Wan-Yuo Guo, MD, PhD^1,2^

^1^ Department of Radiology, Taipei Veterans General Hospital, Taipei, Taiwan

^2.^ National Yang Ming University, School of Medicine, Taipei, Taiwan

^3^ Department of Neurology, Section of Cerebrovascular Disease, Neurological Institute, Taipei Veterans General Hospital, Taipei, Taiwan

^4^ Division of Medical Image, Far Eastern Memorial Hospital, New Taipei City, Taiwan

Correspondence to:

Feng-Chi Chang, MD

Department of Radiology, Taipei Veterans General Hospital

201 Shih-Pai Rd. Sec. 2, Taipei, Taiwan, 11217, R.O.C.

E-mail: fcchang374@gmail.com

Fax: +886-2-28769310

Tel: +886-2-28757031

|  | Case 1 | Case 2 | Case 3 | Case 4 | Case 5 | Case 6 | Case 7 | Case 8 | Case 9 | Case 10 |
| --- | --- | --- | --- | --- | --- | --- | --- | --- | --- | --- |
| Age, years | 80 | 60 | 16 | 71 | 62 | 78 | 72 | 50 | 80 | 85 |
| Sex: male/female | M | M | M | M | M | F | F | M | M | M |
| Risk factors |  |  |  |  |  |  |  |  |  |  |
| Hypertension | Yes | Yes | No | Yes | Yes | Yes | Yes | Yes | Yes | Yes |
| Diabetes | Yes | No | No | Yes | No | Yes | No | Yes | Yes | Yes |
| Hyperlipidemia | No | No | No | No | No | Yes | Yes | No | No | No |
| Smoking | Yes | Yes | No | No | No | No | No | Yes | Yes | No |
| Diagnosis |  |  |  |  |  |  |  |  |  |  |
| Atherosclerosis |  |  |  |  |  |  | Yes |  | Yes |  |
| Dissection | Yes | Yes | Yes | Yes | Yes |  |  |  |  | Yes |
| Dissection in atherosclerosis |  |  |  |  |  | Yes |  | Yes |  |  |
| Time interval between symptoms and MR exam (mon) | 0.7 | 7 | 11 | 0.2 | 30 | 1.5 | 12 | 4 | 0.2 | 0.03 |
| MR findings |  |  |  |  |  |  |  |  |  |  |
| Brain infarcts on MRI |  |  |  |  |  |  |  |  |  |  |
| Nil |  |  |  |  |  |  |  |  |  |  |
| Brainstem | Yes | No | Yes | Yes | Yes | Yes | Yes | Yes | Yes | Yes |
| Cerebellum | Yes | Yes | Yes | Yes | Yes | No | No | Yes | Yes | No |
| Thalamus / Occipital Region | No | Yes | Yes | Yes | No | Yes | Yes | Yes | Yes | Yes |
| Associated other intracranial arterial stenosis | Yes | No | No | No | Yes | Yes | Yes | Yes | Yes | No |
| (Nil/ICA/MCA/ACA) |  |  |  |  |  |  |  |  |  |  |
| Location of stenotic lesions |  |  |  |  |  |  |  |  |  |  |
| Basilar artery |  |  |  | Yes | Yes | Yes |  |  | Yes |  |
| Vertebral artery |  | Yes |  |  |  |  |  |  |  |  |
|  | Case 1 | Case 2 | Case 3 | Case 4 | Case 5 | Case 6 | Case 7 | Case 8 | Case 9 | Case 10 |
| Vertebral to basilar artery | Yes |  | Yes |  |  |  | Yes | Yes |  | Yes |
| Severity of stenotic lesions (%) | 90 | 99 | 90 | 90 | 60 | 70 | 70 | 99 | 80 | 80 |
| Signals of the most stenotic lesions on HR-VWI |  |  |  |  |  |  |  |  |  |  |
| T2WI:high / iso-to low |  |  |  |  |  |  |  |  |  |  |
| high | Yes | No | No | No | Yes | No | No | Yes | Yes | No |
| iso-to low | No | Yes | Yes | Yes | No | Yes | Yes | No | No | Yes |
| T1WI: high / iso-to low |  |  |  |  |  |  |  |  |  |  |
| high | Yes | Yes | Yes | Yes | Yes | Yes | No | Yes | Yes | Yes |
| iso-to low | No | No | No | No | No | No | Yes | No | No | No |
| T1WI + C: strong / faint or no. |  |  |  |  |  |  |  |  |  |  |
| strong | No | No | No | Yes | No | No | No | Yes | No | No |
| faint or no. | Yes | Yes | Yes | No | Yes | Yes | Yes | No | Yes | Yes |
| Restricted diffusion of the stenotic lesions: |  |  |  |  |  |  |  |  |  |  |
| yes / no | Yes | No | No | No | No | No | No | No | No | No |
| Angioplasty and stenting |  |  |  |  |  |  |  |  |  |  |
| Predilatation pressure (atm) | 3 | 4 | 3 | 3.5 | 3.5 | 5 | 7 | 7 | 2 | 3 |
| Stents (self-expandable/balloon-expandable, n=30) |  |  |  |  |  |  |  |  |  |  |
| self-expandable |  |  |  |  |  |  | Yes | Yes | Yes |  |
| balloon-expandable | Yes | Yes | Yes | Yes | Yes | Yes |  |  |  | Yes |
| Technical success | Yes | Yes | Yes | Yes | Yes | Yes | Yes | Yes | Yes | Yes |
| Complications | Yes | No | Yes | No | Yes | No | No | Yes | Yes | No |
|  | Case 1 | Case 2 | Case 3 | Case 4 | Case 5 | Case 6 | Case 7 | Case 8 | Case 9 | Case 10 |
| Outcomes (n=30) |  |  |  |  |  |  |  |  |  |  |
| Follow-up period (mon) | 10 | 60 | 60 | 29 | 36 | 55 | 23 | 1 | 3 | 24 |
| Severe restenosis (≥50%) | No | No | No | No | No | No | No | Yes | No | No |
| Recurrent stroke / symptoms | No | No | No | No | No | No | No | Yes | No | No |
| Mortality | No | No | No | No | No | No | No | Yes | No | No |

|  | Case 11 | Case 12 | Case 13 | Case 14 | Case 15 | Case 16 | Case 17 | Case 18 | Case 19 | Case 20 | Case 21 |
| --- | --- | --- | --- | --- | --- | --- | --- | --- | --- | --- | --- |
| Age, years | 78 | 62 | 76 | 65 | 62 | 58 | 71 | 54 | 73 | 61 | 64 |
| Sex: male/female | M | M | M | M | F | M | M | M | M | M | M |
| Risk factors |  |  |  |  |  |  |  |  |  |  |  |
| Hypertension | No | Yes | Yes | Yes | Yes | Yes | Yes | No | Yes | Yes | Yes |
| Diabetes | Yes | Yes | No | No | No | No | No | No | No | No | Yes |
| Hyperlipidemia | No | No | Yes | Yes | No | No | No | No | No | No | Yes |
| Smoking | Yes | Yes | Yes | No | No | Yes | Yes | Yes | Yes | Yes | Yes |
| Diagnosis |  |  |  |  |  |  |  |  |  |  |  |
| Atherosclerosis |  | Yes |  | Yes | Yes |  | Yes |  | Yes |  | Yes |
| Dissection | Yes |  |  |  |  |  |  |  |  | Yes |  |
| Dissection in atherosclerosis |  |  | Yes |  |  | Yes |  | Yes |  |  |  |
| Time interval between symptoms and MR exam (mon) | 0.03 | 2.5 | 3 | 9 | 2 | 1 | 1 | 12 | 0.2 | 0.1 | 4 |
| MR findings |  |  |  |  |  |  |  |  |  |  |  |
| Brain infarcts on MRI |  |  |  |  |  |  |  |  |  |  |  |
| Nil |  |  |  |  |  |  |  |  |  |  |  |
| Brainstem | Yes | No | Yes | No | Yes | No | Yes | Yes | Yes | Yes | No |
| Cerebellum | Yes | Yes | Yes | Yes | Yes | Yes | No | Yes | Yes | No | No |
| Thalamus / Occipital Region | Yes | No | Yes | Yes | Yes | Yes | Yes | Yes | Yes | No | No |
| Associated other intracranial arterial stenosis | No | Yes | Yes | Yes | Yes | YesYes | Yes | No | Yes | No | Yes |
| (Nil/ICA/MCA/ACA) |  |  |  |  |  |  |  |  |  |  |  |
| Location of stenotic lesions |  |  |  |  |  |  |  |  |  |  |  |
| Basilar artery | Yes | Yes | Yes |  |  | Yes | Yes |  | Yes | Yes | Yes |
| Vertebral artery |  |  |  |  |  |  |  |  |  |  |  |
|  | Case 11 | Case 12 | Case 13 | Case 14 | Case 15 | Case 16 | Case 17 | Case 18 | Case 19 | Case 20 | Case 21 |
| Vertebral to basilar artery |  |  |  | Yes | Yes |  |  | Yes |  |  |  |
| Severity of stenotic lesions (%) | 80 | 85 | 75 | 80 | 90 | 80 | 75 | 85 | 90 | 85 | 80 |
| Signals of the most stenotic lesions on HR-VWI |  |  |  |  |  |  |  |  |  |  |  |
| T2WI:high / iso-to low |  |  |  |  |  |  |  |  |  |  |  |
| high | Yes | No | No | No | No | No | No | No | No | No | No |
| iso-to low | No | Yes | Yes | Yes | Yes | Yes | Yes | Yes | Yes | Yes | Yes |
| T1WI: high / iso-to low |  |  |  |  |  |  |  |  |  |  |  |
| high | No | Yes | Yes | No | No | Yes | No | No | No | Yes | Yes |
| iso-to low | Yes | No | No | Yes | Yes | No | Yes | Yes | Yes | No | No |
| T1WI + C: strong / faint or no. |  |  |  |  |  |  |  |  |  |  |  |
| strong | No | No | No | No | No | No | No | Yes | No | Yes | No |
| faint or no. | Yes | Yes | Yes | Yes | Yes | Yes | Yes | No | Yes | No | Yes |
| Restricted diffusion of the stenotic lesions: |  |  |  |  |  |  |  |  |  |  |  |
| yes / no | No | No | No | No | No | Yes | No | No | No | Yes | No |
| Angioplasty and stenting |  |  |  |  |  |  |  |  |  |  |  |
| Predilatation pressure (atm) | 3 | 4 | 6 | 7 | 6 | 4 | 6 | 5 | 7 | 2 | 6 |
| Stents (self-expandable/balloon-expandable, n=30) |  |  |  |  |  |  |  |  |  |  |  |
| self-expandable | Yes | Yes | Yes | Yes | Yes | Yes | Yes | Yes | Nil | Yes | Yes |
| balloon-expandable |  |  |  |  |  |  |  |  | Nil |  |  |
| Technical success | Yes | Yes | Yes | Yes | Yes | Yes | Yes | Yes | No | Yes | Yes |
| Complications | No | No | No | Yes | No | No | No | No | Yes | No | Yes |
|  | Case 11 | Case 12 | Case 13 | Case 14 | Case 15 | Case 16 | Case 17 | Case 18 | Case 19 | Case 20 | Case 21 |
| Outcomes (n=30) |  |  |  |  |  |  |  |  |  |  |  |
| Follow-up period (mon) | 10 | 48 | 46 | 11 | 40 | 9 | 12 | 20 | 24 | 36 | 16 |
| Severe restenosis (≥50%) | No | No | No | Yes | No | No | No | Yes | Nil | No | Yes |
| Recurrent stroke / symptoms | No | No | No | Yes | No | No | No | Yes | Nil | No | Yes |
| Mortality | No | No | No | Yes | No | No | No | No | Nil | No | No |

|  | Case 22 | Case 23 | Case 24 | Case 25 | Case 26 | Case 27 | Case 28 | Case 29 | Case 30 | Case 31 |
| --- | --- | --- | --- | --- | --- | --- | --- | --- | --- | --- |
| Age, years | 69 | 47 | 46 | 51 | 44 | 52 | 64 | 64 | 51 | 63 |
| Sex: male/female | M | M | M | M | M | M | M | M | M | M |
| Risk factors |  |  |  |  |  |  |  |  |  |  |
| Hypertension | No | No | No | No | No | No | Yes | Yes | Yes | Yes |
| Diabetes | No | No | No | No | No | No | No | No | Yes | No |
| Hyperlipidemia | No | No | No | No | Yes | No | Yes | Yes | No | Yes |
| Smoking | Yes | Yes | No | No | Yes | No | No | No | Yes | Yes |
| Diagnosis |  |  |  |  |  |  |  |  |  |  |
| Atherosclerosis | Yes |  |  |  |  |  | Yes |  |  | Yes |
| Dissection |  | Yes | Yes |  | Yes | Yes |  |  |  |  |
| Dissection in atherosclerosis | Yes |  |  | Yes |  |  |  | Yes | Yes |  |
| Time interval between symptoms and MR exam (mon) | 1 | 0.1 | 0.5 | 0.1 | 0.1 | 0.3 | 0.1 | 0.1 | 24 | 11 |
| MR findings |  |  |  |  |  |  |  |  |  |  |
| Brain infarcts on MRI |  |  |  |  |  |  |  |  |  |  |
| Nil |  |  |  |  |  |  |  |  |  |  |
| Brainstem | No | Yes | No | No | No | No | Yes | Yes | No | No |
| Cerebellum | No | No | Yes | No | Yes | No | No | No | No | No |
| Thalamus / Occipital Region | No | No | Yes | No | No | No | No | No | No | No |
| Associated other intracranial arterial stenosis | No | No | No | No | No | No | No | No | No | Yes |
| (Nil/ICA/MCA/ACA) |  |  |  |  |  |  |  |  |  |  |
| Location of stenotic lesions |  |  |  |  |  |  |  |  |  |  |
| Basilar artery | Yes |  |  |  | Yes |  |  |  | Yes |  |
| Vertebral artery |  |  |  |  |  | Yes | Yes | Yes |  |  |
|  | Case 22 | Case 23 | Case 24 | Case 25 | Case 26 | Case 27 | Case 28 | Case 29 | Case 30 | Case 31 |
| Vertebral to basilar artery |  | Yes | Yes | Yes |  |  |  |  |  | Yes |
| Severity of stenotic lesions (%) | 90 | 99 | 95 | 80 | 85 | 70 | 85 | 99 | 70 | 99 |
| Signals of the most stenotic lesions on HR-VWI |  |  |  |  |  |  |  |  |  |  |
| T2WI:high / iso-to low |  |  |  |  |  |  |  |  |  |  |
| high | No | No | Yes | No | No | Yes | No | No | No | No |
| iso-to low | Yes | Yes | No | Yes | Yes | No | Yes | Yes | Yes | Yes |
| T1WI: high / iso-to low |  |  |  |  |  |  |  |  |  |  |
| high | No | Yes | Yes | No | Yes | Yes | No | Yes | No | No |
| iso-to low | Yes | No | No | Yes | No | No | Yes | No | Yes | Yes |
| T1WI + C: strong / faint or no. |  |  |  |  |  |  |  |  |  |  |
| strong | No | Yes | Yes | Yes | No | Yes | No | Yes | No | No |
| faint or no. | Yes | No | No | No | Yes | No | Yes | No | Yes | Yes |
| Restricted diffusion of the stenotic lesions: |  |  |  |  |  |  |  |  |  |  |
| yes / no | Yes | Yes | Yes | No | No | Yes | No | No | No | No |
| Angioplasty and stenting |  |  |  |  |  |  |  |  |  |  |
| Predilatation pressure (atm) | 7 | 3 | 3 | 5 | 3 | 3 | 7 | 5 | 7 | 7 |
| Stents (self-expandable/balloon-expandable, n=30) |  |  |  |  |  |  |  |  |  |  |
| self-expandable | Yes | Yes | Yes |  |  | Yes |  | Yes | Yes | Yes |
| balloon-expandable |  |  |  | Yes | Yes |  | Yes |  |  |  |
| Technical success | Yes | Yes | Yes | Yes | Yes | Yes | Yes | Yes | Yes | Yes |
| Complications | Yes | Yes | No | No | No | No | No | No | Yes | No |
|  | Case 22 | Case 23 | Case 24 | Case 25 | Case 26 | Case 27 | Case 28 | Case 29 | Case 30 | Case 31 |
| Outcomes (n=30) |  |  |  |  |  |  |  |  |  |  |
| Follow-up period (mon) | 13 | 0.2 | 26 | 13 | 22 | 16 | 8 | 8 | 6 | 7 |
| Severe restenosis (≥50%) | No | No | No | No | No | No | No | No | Yes | No |
| Recurrent stroke / symptoms | No | No | No | No | No | No | No | No | No | No |
| Mortality | No | Yes | No | No | No | No | No | No | No | No |
